# Coral reef islands can accrete vertically in response to sea level rise

**DOI:** 10.1126/sciadv.aay3656

**Published:** 2020-06-10

**Authors:** Gerd Masselink, Eddie Beetham, Paul Kench

**Affiliations:** 1 School of Biological and Marine Sciences, University of Plymouth, Plymouth PL4 8AA, UK.; 2 School of Environment, University of Auckland, Private Bag, Auckland 92010, New Zealand.; 3 Department of Earth Sciences, Simon Fraser University, Burnaby, BC V5A 1S6, Canada.

## Abstract

Increased flooding due to sea level rise (SLR) is expected to render reef islands, defined as sandy or gravel islands on top of coral reef platforms, uninhabitable within decades. Such projections generally assume that reef islands are geologically inert landforms unable to adjust morphologically. We present numerical modeling results that show reef islands composed of gravel material are morphodynamically resilient landforms that evolve under SLR by accreting to maintain positive freeboard while retreating lagoonward. Such island adjustment is driven by wave overtopping processes transferring sediment from the beachface to the island surface. Our results indicate that such natural adaptation of reef islands may provide an alternative future trajectory that can potentially support near-term habitability on some islands, albeit with additional management challenges. Full characterization of SLR vulnerability at a given reef island should combine morphodynamic models with assessments of climate-related impacts on freshwater supplies, carbonate sediment supply, and future wave regimes.

## Teaser

Coral reef islands are morphodynamically resilient landforms that can adjust to sea level rise through vertical accretion.

## INTRODUCTION

Coral reef islands, defined as sandy or gravel islands on top of coral reef platforms, are among the most vulnerable coastal environments to sea level rise (SLR) (*1*–*3*). Prominent literature on this topic has focused on the consequences of increased coastal flooding on island habitability using hydrodynamic modeling approaches, and the current thinking is that these islands will become uninhabitable in the next few decades (*4*). This has generally resulted in a binary consideration of adaptation solutions of either structural defense or the exodus of island communities, with considerably less attention on options that build resilience. Our paper redresses that balance and uses a process-based numerical model that simulates the morphological response of reef islands to SLR. Model results demonstrate that islands can adjust vertically (building upward) and horizontally (migrating landward) via the process of “rollover” (*5*), which involves the transport of sediment from the front of the island to the top and the back of the island via overtopping and overwash processes. The research we present is a critical development for understanding and managing the impact of SLR on atoll island communities and will provide a benchmark for future investigations that need to account for the morphodynamic nature of atoll islands.

SLR and associated climatic change are among the greatest environmental threats to the continued sovereignty and livelihood of midocean island states (*3*, *6*, *7*). Low-lying coral reef islands are considered most at risk, with rising sea levels and increased storminess expected to physically destabilize islands (*1*, *2*, *8*) and increase the frequency and magnitude of flooding to such an extent they will render islands uninhabitable in the coming decades (*4*). However, such existing assessments of physical island vulnerability are based on assumptions that islands are geologically static and will simply drown as sea level increases (*1*, *4*, *9*). Under these environmental scenarios, “island loss” has become normalized, sociopolitical debate has focused on concerns of human security (*7*, *10*), and the future trajectory of island communities has been reduced to a binary of protection by means of coastal defences (*11*, *12*) or relocation (*13*). However, there is growing evidence that islands are geologically dynamic features that can adjust to changing sea level and climatic conditions (*14*, *15*). The loss of coral reef islands due to climate change is therefore not a fait accompli, and a more optimistic prognosis may exist for island nations (*16*).

Reef islands are wave-built accumulations of carbonate sediment derived from the breakdown of calcium carbonate–secreting organisms that dwell on the adjacent coral reef systems. The location, planform configuration, size, and elevation of islands reflect both the interaction of oceanic swell with reef structures and the availability and grade of sediment for island building. Recent studies have demonstrated that the planform configuration of reef islands can be modified in response to changing wave regimes and sea level from event to centennial time scales (*17*, *18*). Furthermore, studies reveal a dominant trend of island expansion on reef surfaces over the past half century, a period of documented SLR (*19*–*22*).

Despite these recent advances in understanding the morphological behavior of islands, several uncertainties remain. First is whether recently documented morphodynamic responses in island planform provide analogs for future island change, particularly as the magnitude and rates of SLR are expected to increase (*23*–*25*). Second is the extent to which morphodynamic processes modify island elevation and buffer the impacts of rising sea levels and increased flood risk (*4*). To date, topographic changes in island surfaces have not been resolved. Addressing the ability of geomorphic processes to alter elevation is critical, as it is island elevation that influences the frequency and magnitude of wave-driven overwash and island flooding and, consequently, risk to island communities (*26*). A further and fundamental gap in the assessment of island response to sea level change is the lack of a robust process-based analysis of island morphodynamics that can project future morphological trajectories of islands. This lack of reliable future predictions is a major constraint in considering and developing more grounded adaptation strategies. However, recent physical model experiments of island change (*15*) and advances in physics-based numerical modeling of sediment transport and morphodynamic change have substantially enhanced the potential to explore how SLR will affect atoll island morphology and future susceptibility to flooding and erosion.

Here, we present the first application of a numerical model, validated using a small-scale physical model, to simulate the morphological adjustment of reef islands to rising sea level. Results highlight the morphological adjustments of islands, including elevation, and reveal characteristic modes of island response. The findings are used to explore sensitivities of islands to changing sea level dynamics and consider adaptation responses.

## RESULTS

### Physical model and numerical validation

**Fig. 1:**
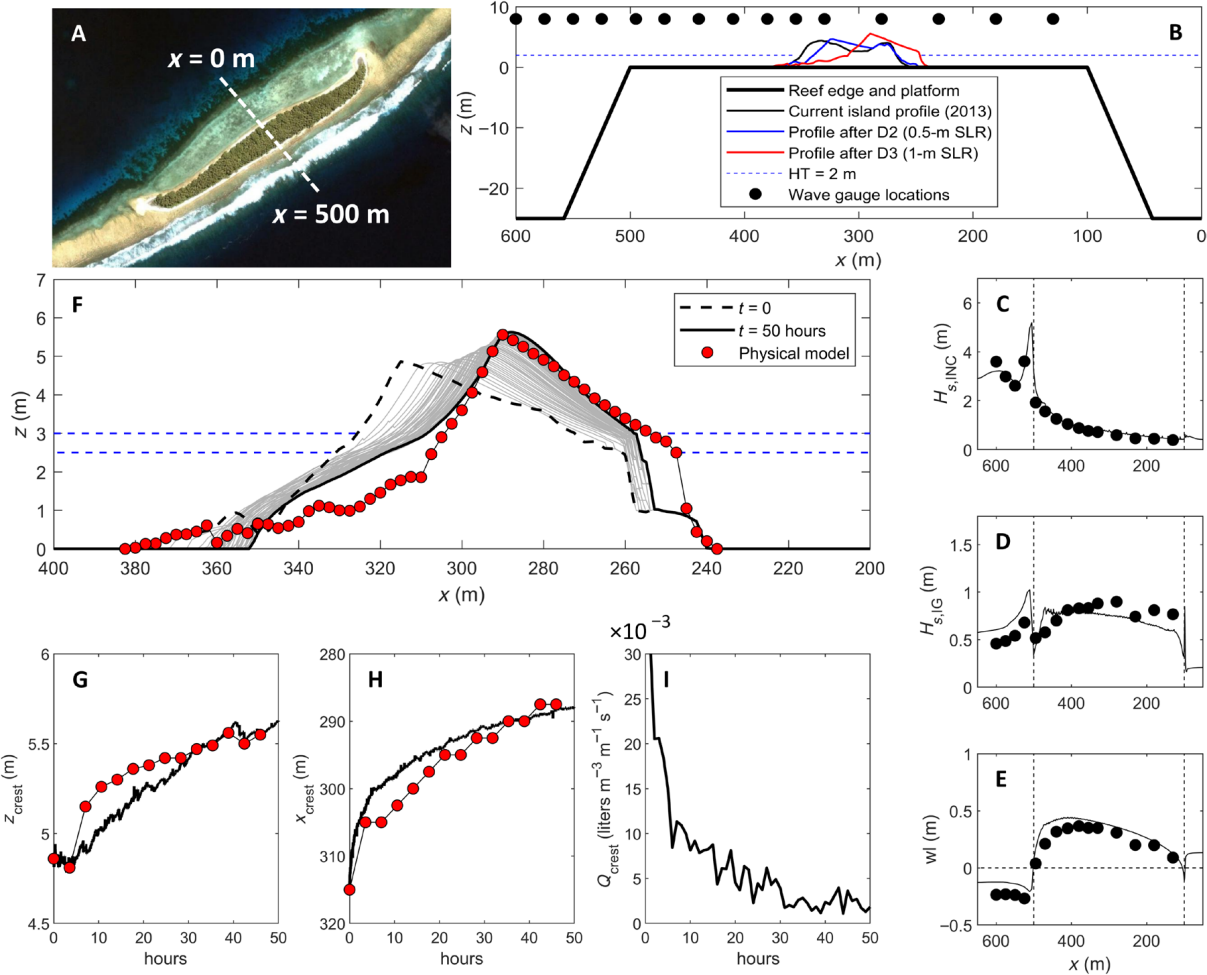
Reef island response to SLR. (**A**) Aerial photograph of Fatato, Funafuti atoll, Tuvalu; white dashed line indicates central profile line. (**B**) Experimental setup in the physical and numerical model. (**C** to **E**) Physical model data (black circles) and numerical model results (black line) of (C) incident significant wave height *H*_s,INC_, (D) infragravity significant wave height *H*_s,IG_, and (E) mean water level *wl* for a run with *H*_s_ = 4 m, *T*_p_ = 9.9 s, and *h*_reef_ = 1 m. Vertical black dashed lines represent the reef platform edge. (**F**) Measured and modeled reef island morphology after 50 hours (*H*_s_ = 4 m and *T*_p_ = 9.9 s in the numerical model; representing 7 hours, *H*_s_ = 0.08 m, and *T*_p_ = 1.3 s in the physical model) with sea level raised from *h*_reef_ = 2.5 m to *h*_reef_ = 3 m for the optimal combination of the relevant model parameters (*D*_50_ = 14 mm; *K* = 0.005 m s^−1^; ϕ = 25^o^). (**G** to **I**) Measured (red circles) and modeled (black line) time series of (G) island crest elevation *z*_crest_, (H) island crest position *x*_crest_, and (I) overwash discharge *Q*_crest_. The 1:50 scale physical experiment results are plotted at the prototype scale.

Results of scaled (1:50) physical modeling of the response of Fatato Island, Funafuti atoll, Tuvalu (Fig. 1A), to two consecutive 0.5-m step increases in sea level under extreme wave conditions (*H*_s_ = 4 m; *T*_p_ = 9.9 s at prototype scale; see the “Physical modeling” section) indicate that reef islands can physically adapt to SLR and maintain a positive freeboard (defined as the elevation difference between the island crest and sea level). The island crest (i.e., highest point) accreted vertically by 0.6 m during the second 0.5-m increase in sea level while retreating lagoonward by 25 m (Fig. 1, B and F to H) (*15*). The physical mechanism for this adjustment is wave overtopping, where run-up exceeds the crest of the island, driven by SLR increasing wave height and water level at the shoreline. Wave overtopping effectively transfers sediment from the nearshore and beachface to the island crest and surface and is the primary mechanism for vertical island accretion. The net result is a morphodynamic rollover response similar to that identified on gravel barrier systems (*5*) and in previous analytical modeling studies specific to atoll islands (*27*).

Morphodynamic changes measured in the wave flume were replicated with high skill in the phase-resolving model XBeach-G [(*28*); see the “Numerical modeling” section], providing the first physically evaluated numerical modeling platform to explore island adjustment under different sea level and environmental scenarios. The ability of the numerical model to reproduce wave transformation processes was previously demonstrated (Fig. 1, C to E) (*29*). Multiple combinations of key input parameters (sediment size *D*_50_; hydraulic conductivity *K*; and sediment transport phase angle φ) were used to drive the numerical model, and outputs were compared to the morphodynamic change observations from the physical model (see Table 2). The best results were obtained with *D*_50_ = 14 mm, *k* = 0.005 m s^−1^, and φ = 25° (Fig. 1G), where the Brier skill score (BSS), the crest level adjustment, and the crest migration distance were used as key model performance indicators. Numerical modeling outputs also provide fresh insight into the role of crest discharge associated with overtopping in driving morphological change. Substantial crest discharge (*Q*_crest_ > 0.01 m^3^ m^−1^ s^−1^ or >10 liters m^−1^ s^−1^) and island adjustment occur initially, as the island responds rapidly to a forced step increase in sea level. However, as the island adjusts vertically in response to a higher sea level state, there is a compensating reduction in crest discharge (Fig. 1I). Perhaps unexpectedly, the island adjustment is accomplished by relatively modest overwash dynamics: the mean overwash volume across the island crest for *t* = 10 to 50 hours of the simulation was 0.004 m^3^ m^−1^ s^−1^, and the mean of the hourly maximum overwash depth at the same location and over the same time period was 0.12 m.

### Overwash versus overtopping

**Fig. 2:**
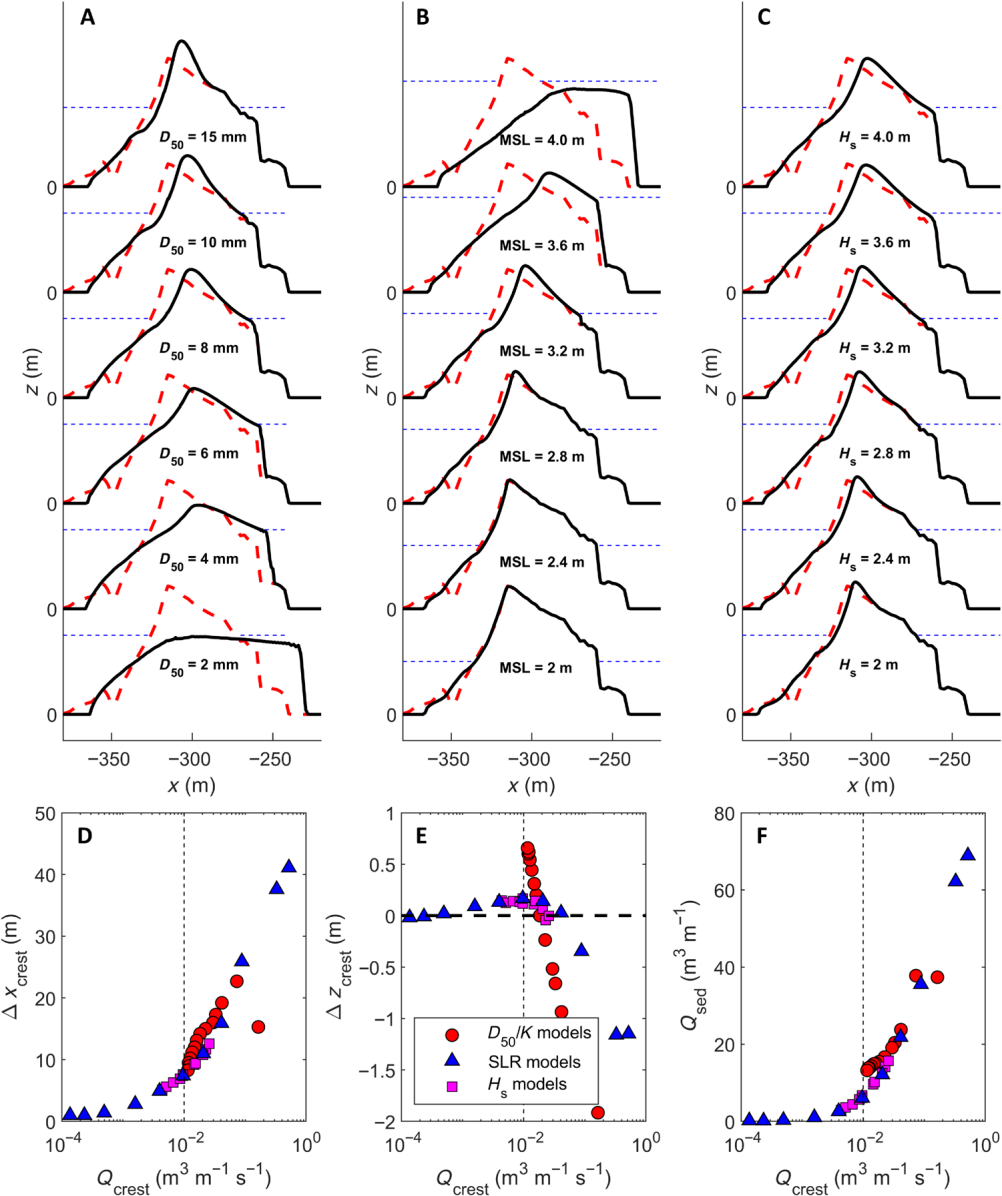
Results of 3-hour numerical model simulations of reef island response to SLR. (**A** to **C**) Sensitivity of island response to different forcing scenarios and environmental conditions: (A) island sediment size (*D*_50_) and associated hydraulic conductivity (*K*), (B) sea level (MSL), and (C) offshore significant wave height (*H*_s_). Red dashed line and black solid line represent profile at the start and end of the 3-hour model run. (**D** to **F**) Correlation between overtopping discharge averaged over the full 3-hour model run across the moving island crest (*Q*_crest_) and morphological response parameters: (D) change in island crest position (Δ*x*_crest_), (E) change in crest elevation *(*Δ*z*_crest_), and (F) sediment discharge across the crest (*Q*_sed_). The vertical dashed line in (D) to (F) represents *Q*_crest_ = 0.001 m^3^ m^−1^ s^−1^ or 10 liters m^−1^ s^−1^.

Additional numerical modeling simulations show how morphodynamic adjustment varies under different environmental conditions, including island sediment size, the magnitude of SLR, and offshore wave height (see the “Numerical modeling” section). Results indicate that water discharge across the island crest is the key control on the magnitude and style of the resulting morphological adjustment (Fig. 2). In all tested scenarios, overtopping drives moderate crest discharge and is associated with crest buildup and island accretion [“overtopping” scenario; cf. (*5*)]. In contrast, higher discharges are associated with overwashing episodes and result in crest flattening [“overwashing” scenario; cf. (*5*)]. Increasing crest discharge from zero initially promotes overtopping and enhances vertical accretion at the island crest, with the maximum increase in positive freeboard associated with a mean crest discharge of 0.01 to 0.02 m^3^ m^−1^ s^−1^, before higher discharge magnitudes result in crest lowering (Fig. 2E).

A threshold crest discharge of c. 0.01 m^3^ m^−1^ s^−1^ therefore differentiates constructive overtopping events from destructive overwashing events, although overwashing does promote accretion at leeward locations. This threshold is similar to the engineering design threshold for overtopping discharge that will cause damage or erosion on rubble mound breakwaters and was typically associated with maximum water depths of 0.2 to 0.4 m (fig. S2). These results have important implications for the expected direction and pace of change of predominantly gravel versus sand islands and the nature of island response in high-energy or storm-exposed settings. For an island to accrete vertically in response to future SLR, conditions that promote overtopping need to occur more frequently than conditions that produce overwashing and crest lowering.

### Island adjustment to SLR

**Fig. 3:**
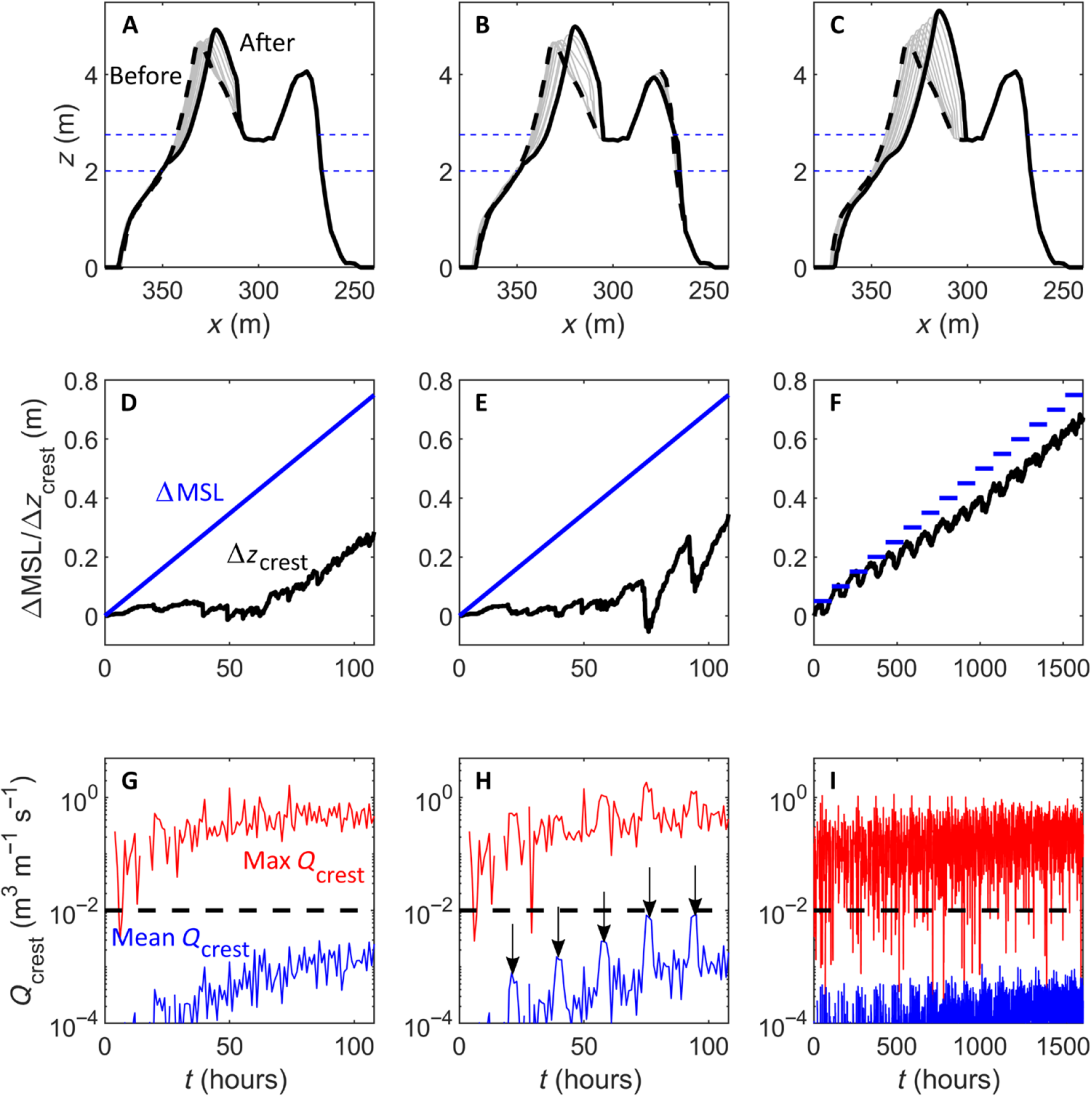
Results of 0.75-m SLR simulations. (**A** to **C**) Island adjustment to (A) 0.75 m of gradual SLR over 108 hours and typical annual storm wave conditions, (B) 0.75 m of gradual SLR over 108 hours with typical annual storm wave conditions and extreme wave perturbations, and (C) fifteen 0.05-m step changes in sea level over a total of 1620 hours and typical annual storm wave conditions. The horizontal blue dashed lines in (A) to (C) represent the sea level at the start and end of the simulation. (**D** to **F**) Change in island crest elevation (Δ*z*_crest_) and sea level (ΔMSL) relative to the start of each simulation for model simulations shown in (A) to (C), respectively. (**G** to **I**) Hourly averaged and hourly maximum water discharge across the moving island crest *Q*_crest_ for model simulations shown in (A) to (C), respectively, with the dashed line indicating *Q*_crest_ = 0.01 m^3^ m^−1^ s^−1^. The vertical arrows in (H) represent the 3-hour episodes of extreme wave action (*H*_s_ = 3 to 3.8 m).

Three numerical modeling scenarios were used to explore island adjustment to an SLR of 0.75 m, the global average increase predicted for 2100 (*23*). Informed by wave climate analysis (*30*), offshore wave height alternated between moderate (*H*_s_ = 2.2 m) and annual maximum (*H*_s_ = 2.6 m) every hour, with water level at each time step representing the magnitude of SLR at spring high tide height (see the “SLR simulations” section in Materials and Methods). Under these conditions, when exposed to 0.75 m of gradual SLR over 108 hours, the island crest adjusted by accreting vertically (0.29 m) and retreating landward (9 m), resulting in a net loss in freeboard of 0.46 m (Fig. 3, A and D). When repeating this simulation, but with 3-hour perturbations of extreme wave height every 15 hours (increasing from *H*_s_ = 3 m to *H*_s_ = 3.8 m; see the “SLR simulations” section, Materials and Methods), a similar pattern of island change occurred, with the crest generally building up during the dominant overtopping conditions but lowering during the most extreme wave forcing, resulting in overwash conditions, and eventually producing a very similar net loss in island freeboard of 0.41 m and a landward retreat of 11 m (Fig. 3, B and E). Both 108-hour-long simulations indicate that hardly any island adjustment occurred during the first 0.4 m of SLR due to very limited overtopping. This was followed by rapid morphological adjustment, with the rate of vertical island building similar to the rate of SLR, indicating that the island is attempting to keep pace with the rising sea level. However, the gradual but substantial increase in overtopping discharge during the second half of both simulations is linked to the island’s decreasing freeboard (Fig. 3, G and H). The overwash dynamics during the second part of both simulations was characterized by an average discharge of 0.001 to 0.002 m^3^ m^−1^ s^−1^, and the mean of the hourly maximum overwash depth at the same location and over the same time period was 0.2 m.

To explore whether a morphodynamic equilibrium to SLR can be achieved, the island was exposed to fifteen 0.05-m step increases in sea level for 108 hours each (see the “SLR simulations” section, Materials and Methods). This longer simulation produced substantially different results, with initial crest adjustment up to SLR = 0.2 m exceeding the magnitude of SLR. However, as SLR continued, the accreting island crest started to lag behind SLR, with a final net accretion of 0.68 m at SLR = 0.75 m, indicating a 0.07-m loss in freeboard, accompanied by a lagoonward retreat of 16.4 m (Fig. 3, C and F). Throughout the long simulation, hourly averaged discharge remained an order of magnitude lower than the shorter 108-hour scenarios (see table S1). Discharge increased during the longer simulation but stabilized after 1000 hours and remained well below the threshold for overwash and crest lowering (Fig. 3I). The overwash dynamics during the second part of the long simulation was characterized by an average discharge of 0.0002 m^3^ m^−1^ s^−1^, and the hourly maximum overwash depth at the same location was at all times less than 0.1 m.

Temporary and minor lowering of the island crest occurred in all SLR simulations, always during a single set of large waves (not shown), but the only substantial occurrences of island lowering occurred when the hourly averaged crest discharge exceeded 0.01 m^3^ m^−1^ s^−1^ during sustained high wave activity (*H*_s_ = 3.6 and 3.8 m; Fig. 3H). These results indicate that reef islands can physically adjust to SLR when overtopping dominant conditions promote crest buildup and can recover from periodic overwash events that lower the crest. Results also suggest that the rate of future sea level change will be critical in determining the balance between overtop and overwash dominant regimes, something also suggested by studies of the long-term evolution of gravel barriers due to SLR (*5*).

## DISCUSSION

Our numerical model simulations, validated against small-scale physical model tests, indicate that reef islands will undergo physical transformations in response to SLR and can maintain island surfaces above sea level. Notably, we present the first process-based numerical model simulations of future island change that highlight lateral displacement of shorelines and vertical building of the island crest and island surface. These adjustments are mediated through island rollover, driven by overtopping and overwash processes, and a tentative threshold separating these two regimes for our modeled gravel barrier subjected to wave conditions of *H*_s_ = 2–4 m is a mean overwash discharge across the island crest of 0.01 m^3^ m^−1^ s^−1^ associated with maximum overwash depths of 0.2 to 0.4 m. Our results further indicate that the magnitude and pace of change will be dependent on both the rate of SLR and changing wave regimes. These modeled trajectories of island dynamics are consistent with modes of island change observed in recent studies throughout the Indo-Pacific (*17*, *19*, *20*–*22*).

The morphological modeling approach adopted here considers coral reef island response to climate change only as a result of rising sea level. However, increased ocean water temperature is expected to increase the intensity of tropical storms, resulting in enhanced coastal flooding (*31*), thereby accelerating the rollover process identified in this study, and also has substantial adverse effects on the health of coral reef systems that may modify carbonate sediment production regimes that contribute to island building and maintenance (*32*). In addition, island habitability is not only a function of island freeboard it also depends on the island planform area, which, without sediment input from the reef structure, may reduce as a result of rollover. Storlazzi *et al.*(*4*) have demonstrated that enhanced coastal flooding due to SLR is expected to lead to increased contamination of the freshwater aquifer, where they occur, a process not accounted for in the present numerical modeling approach. It is also important to emphasize that the reef island modeled here is made of gravel, and because of the reduced mobility and increased hydraulic conductivity of gravel compared with that of sand, it could be argued that gravel islands may be particularly responsive and able to keep up with rising sea level. Notwithstanding a modeling approach that only considers the morphodynamic impacts of SLR and the complex set of factors that influence island habitability (*33*–*35*), our results confirm recent assertions that the physical foundations for island communities may persist (*21*). Compared with a static reef island model, the vertical buildup of island elevation by overwash processes modeled here can also offset the increase in future flood risk due to SLR. However, our results also indicate that communities are likely to be confronted with ongoing and escalating rates of island physical change that will stress populations and require careful consideration of the full spectrum of adaptation strategies.

**Fig. 4:**
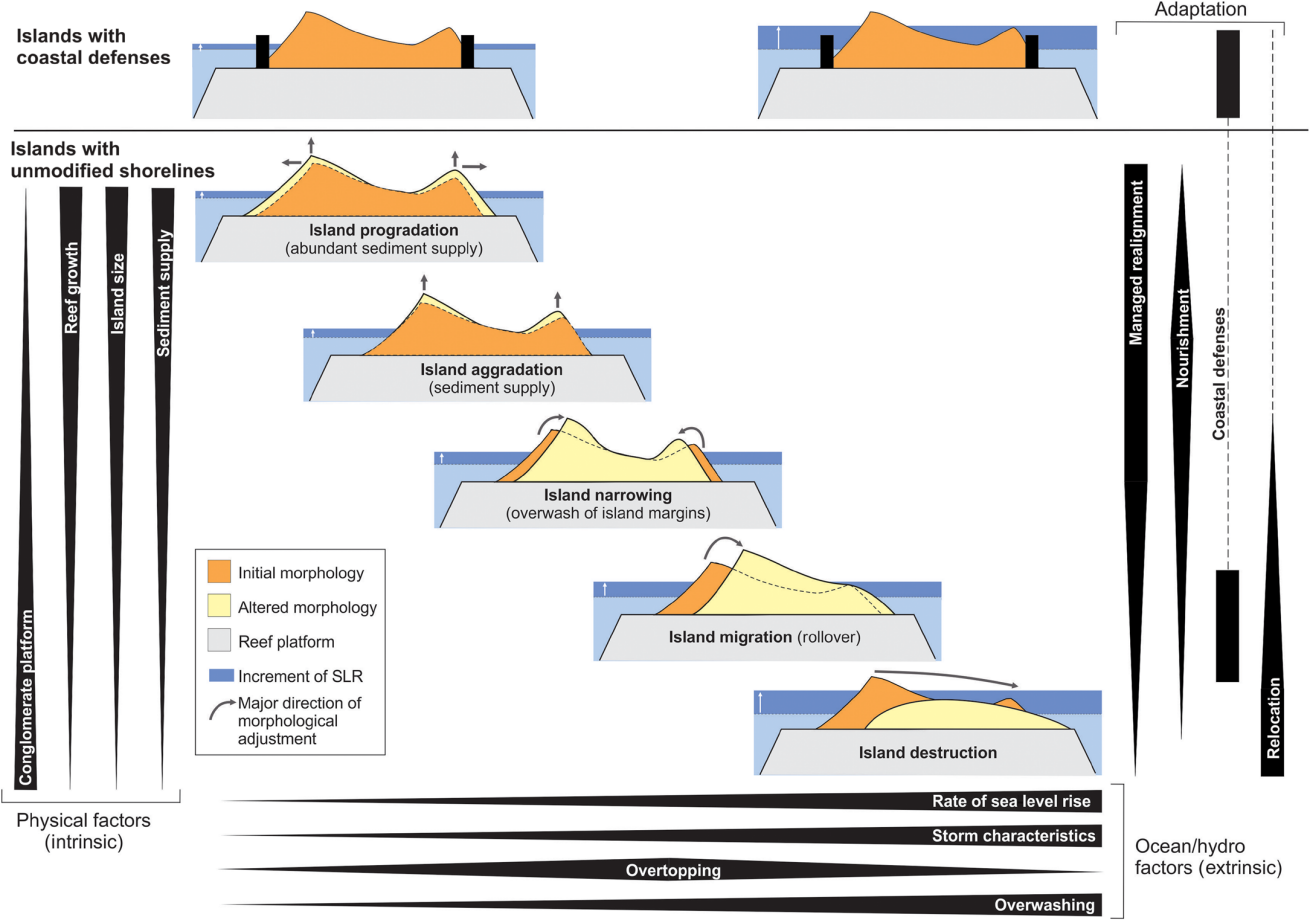
Conceptual diagram of reef island morphological adjustment to future SLR under different environmental and management scenarios. Island response is driven by extrinsic factors (rate of SLR, storm characteristics, and overtopping/overwashing balance) and controlled by intrinsic factors (presence/absence of conglomerate platform beneath the island, reef growth, size of the island, and sediment supply). The most appropriate adaptation strategy (managed realignment, nourishment, coastal defense, and relocation) to deal with island change is strongly determined by the type of island response to SLR. For example, an island that is narrowing, but maintaining freeboard, could benefit more from nourishment than coastal defense. If an island is already completely defended, preventing overtopping and overwashing, the only way to maintain habitation is upgrading the coastal defenses (or relocation). The width of the black bars represents the magnitude/importance/relevance of the factor in question.

Our analysis provides an empirical basis to help inform appropriate adaptation pathways in island nations, with continued habitation of islands underpinning the majority of these approaches (Fig. 4). The simulated morphodynamic trajectories suggest a cascade of responses is likely, beginning with island keep-up and marginal island narrowing under slower rates of SLR and dominant overtopping regimes. Faster-paced lateral migration of islands and increased reduction in freeboard are projected under faster rates (and greater magnitudes) of SLR and higher wave regimes, producing dominant overwash regimes (Fig. 4). In the most extreme cases, dominant overwashing forces loss of freeboard and rapid rollover of island sediment reservoirs. This cascade of morphological changes supports recent studies (*21*, *36*) that indicate that physical responses are likely to vary between islands, reflecting differences in antecedent condition (e.g., sedimentary fabric and abundance, island size, and presence/absence of conglomerate platform) and environmental boundary conditions (storm wave climate and rate of SLR). Such differences in morphodynamic behavior present the opportunity to develop nuanced adaptation solutions in different island settings, rather than adopt a one-solution approach that ultimately results in island abandonment and relocation (*10*). Islands with artificial shoreline defenses compromise the ability of shorelines to undergo natural adjustment to changes in the process regime and lock communities into hard structural solutions and a maladaptive dependency. Under extreme scenarios of change, islands may become uninhabitable, and community relocation and structural solutions may become the only alternatives. However, between the binary outcomes—hold the line and community migration—exist a suite of alternate solutions that reflect the dynamic nature of island change and allow planning and soft engineering strategies. Furthermore, given the progressive nature of island transformations, the suite of options provide opportunities for adaptive planning pathways to be developed at the island scale (*37*), which allows resources to be deployed in a more efficient manner and avoid maladaptive interventions (*38*).

The pursuit of alternate adaptation pathways does not negate the need to pursue ongoing mitigation action to curtail future SLR and climatic changes on small island nations. However, morphodynamic modeling provides a basis to resolve island-specific trajectories of change to underpin the development of adaptation strategies that may extend the duration of habitation of these islands to at least more than several decades. Future morphodynamic modeling of reef island response to SLR must not only explore further SLR and wave conditions but also need to incorporate the different environmental factors, such as island morphology, reef platform adjustment, and sediment supply.

## MATERIALS AND METHODS

The primary goal of this investigation was to use a process-based numerical model to investigate the response of coral reef islands to SLR. First, we used the results of a small-scale laboratory experiment to validate the phase-resolving version of the XBeach numerical model. Second, we used the validated model to explore the sensitivity of island response to different forcing conditions (waves and sea level), controlling factors (sediment size and hydraulic conductivity), and sediment transport formulations. Third, we investigated the discharge of water flowing over the crest of the island under energetic wave and high sea level conditions to distinguish between overtopping (leading to rising of the island crest) and overwash (leading to lowering of the island crest). Fourth, we conducted several lengthy sea level simulations to model the response of reef islands to a 0.75-m SLR.

### Physical modeling

Laboratory experiments were undertaken in a wave flume (length = 20 m, width = 0.6 m, depth = 1 m) at the COAST (Coastal Ocean and Sediment Transport) Lab (University of Plymouth, UK; https://www.plymouth.ac.uk/research/institutes/marine-institute/coast-laboratory). The laboratory reef platform and island model were constructed to a 1:50 scale, and the reef platform and island dimensions were based on topographic profiles of Fatato Island (fig. S1 and Fig. 1A). The horizontal reef platform (8 m by 0.6 m) was constructed from marine plywood and was located 0.47 m above the flume floor, providing a 24° forereef and backreef slope. A thin layer of fine sand was glued onto the marine ply to replicate the roughness of the reef platform. The oceanward reef crest was located 9 m from the face of the wave paddle.

The “gravel island” emplaced on the reef platform was based on the “double-ridged” topographic profile through the center of the existing island of Fatato that was measured in 2013 (Fig. 1B). The highest ridge (*z* = 4.65 m) is found on the ocean side of the island and the lowest ridge (*z* = 4.05 m) on the lagoon side, reflecting differing levels of wave exposure and run-up. This double-ridge morphology is a common characteristic of atoll reef islands (*19*). A wooden template reflecting the real island cross-shore profile was used to manually shape the island in the physical model. The ocean-facing shoreline of the island was located 2.4 m from the reef crest. The island was formed out of fine sand (median *D*_50_ = 0.35 mm), which geometrically scaled to an equivalent grain size of 17.5 mm, comparable to the lower end of the sediment found on Fatato. Using 0.35-mm sediment, the corresponding Shields and Rouse numbers scaled at c. 1:2, depending on the selected wave forcing and water depth. Waves simulated during all experiments were produced by an absorbing piston paddle. All wave conditions were irregular and generated using a JONSWAP (Joint North Sea Wave Project) wave steering signal and represent the most energetic wave conditions that can be experienced at Fatato. Reef platform water levels were recorded using 15 capacitance wire wave probes (Fig. 1B) at a frequency of 32 Hz.

Two sets of experiments were undertaken. First, wave transformation across the reef platform, without an island present, was examined in three experiments (test series A to C) to study wave transformation processes and ensure that the wave conditions in the physical model are consistent with those occurring in reef environments (results of one of the forcing conditions are shown in Fig. 1, C to E). The significant wave height *H*_s_, peak wave period *T*_p_, and reef water depth *h*_reef_ for these hydrodynamic runs without an island are listed in table S2. Each run with unique wave conditions lasted for 12 min, and data analysis was conducted over the last 10 min to allow for 2-min “spin-up” time. Only the results for the most energetic wave condition and midtide water level are presented here; results for the other runs have been published previously (*29*).

Second, island morphological response to changing sea level was examined in test series D, as outlined in table S3. At the start of this series of runs, the scaled island was constructed on the reef platform, and the response of the island to exposure to extreme wave conditions with a short wave period was investigated. Island morphology was documented every 0.5 hour (representing 3.5 hours at the prototype) along the central profile using a laser beam profiler, and a number of morphometric parameters were extracted from the profile data (for this paper, elevation and position of the crest of the island, *z*_crest_ and *x*_crest_, respectively). During the first run D1, which lasted for 1.5 (10.6) hours, the sea level was set to high tide level [*h*_reef_ = 0.02 (1) m], and only very minor morphological changes were recorded. For the second run D2, the sea level was raised by 0.01 (0.5) m, and the island was exposed to the same wave conditions for another 1.5 (10.6) hours. Frequent overwashing resulted in substantial morphological change, and the resulting island profile is shown in Fig. 1B (red line). For the final run D3, the sea level was raised yet another 0.01 (0.5) m to represent a total SLR of 0.02 (1) m, and this run lasted for 7 (49.5) hours, again with the same extreme wave conditions. The island profile at the end of this long run is also shown in Fig. 1B (blue line). It is the time evolution of run D3 that was used for numerical model validation.

### Numerical modeling

The response of coral reef islands to SLR was numerically modeled using XBeach-G, originally developed to model the response of gravel barriers to extreme storm events (*28*). This model is based on the widely used XBeach model, which is an open-source, two-dimensional numerical model in the horizontal plane (2DH) that solves horizontal equations for wave propagation, long waves and mean flow, sediment transport, and morphological change (*39*). XBeach has two main modes: (i) nonhydrostatic (XB-NH), which resolves all wave motions (short-wave resolving), and (ii) surfbeat (XB-SB), which resolves motions on the scale of wave groups but treats short-wave motions in a phase-averaged manner (short-wave averaged). The more computationally demanding XB-NH mode has been used in the development of XBeach-G, as incident wave motion needs to be resolved for simulating swash and overwash processes on steep profiles, such as found on gravel and coral beaches. XB-NH computes depth-averaged flow due to waves and currents using the nonlinear shallow water equations and includes a nonhydrostatic pressure correction, which is derived in a manner similar to a one-layer version of the SWASH (Simulating WAves till SHore) model (*40*).

XBeach-G is a 1DH model, so longshore sediment transport processes are ignored. This is obviously a simplification of the processes involved in island response to SLR; however, wave flume experiments (2D) such as those described in the previous section have also been conducted in a wave basin (3D). Although substantial longshore effects were observed along the flanks of the island placed in the wave basin, the morphological response across the center of the island, namely, rollover, was virtually identical to that observed in the wave flume (*41*), providing justification for the use of a 1DH morphodynamic model.

Compared to the XB-NH model, XBeach-G includes two modifications (*28*): (i) inclusion of swash-groundwater interactions to account for swash infiltration and (2) implementation of sediment transport equations specific to gravel (bedload only). Groundwater interactions are accounted for using a Darcy approach, modified for turbulent groundwater flow, and sediment transport is modeled using the following set of equations

$$q_{s}=12(\theta -0.05)\sqrt{\theta }\sqrt{(\frac{\rho_{s}-\rho }{\rho }){\mathrm{gD}}_{50}^{3}}$$ (1)

where *q*_s_ is the volumetric sediment transport rate (m^3^ s^−1^), θ is the Shields parameter (−), ρ and ρ_s_ are the density of water and sediment (kg m^−3^), respectively, and *D*_50_ is the median grain diameter (m). The Shields parameter is adjusted for bed slope effects

$$\theta =\frac{u_{*}^{2}}{(\frac{\rho_{s}-\rho }{\rho }){\mathrm{gD}}_{50}}\text{cos}(\beta )(1\pm \frac{\text{tan}(\beta )}{\text{tan}(\phi )})$$ (2)

where u* is the friction velocity (m s^−1^), β is the bed angle (°), and ϕ is the angle of repose of the sediment (°). To account for boundary layer expansion and contraction in the swash, the pressure gradient effects, and the presence of turbulent fronts, following (*42*), the friction velocity is computed using the approximation

$$u_{*}=\sqrt{\frac{f_{s}}{2}}(\text{cos}(\varphi )u+\frac{T_{m-1,0}}{2\pi }\text{sin}(\varphi )\frac{\delta u}{\delta t})$$ (3)

where *f*_s_ is the user-defined sediment friction factor in the order of 0.01 (−), *T*_*m*−1,0_ is the offshore spectral period based on the first negative moment of the energy spectrum (s), and φ is a user-defined phase lag angle in the order of 30°. The phase angle φ in this approach is critical as it represents the phase lag between the free-stream velocity and the bed shear stress, and values larger than 0 increasingly promote the onshore transport of sediment. The equation was specifically developed to model horizontally asymmetric wave motion (such as in surf zone bores and overwash), which is known to drive sediment onshore.

For all modeling runs, the waves were forced from a depth of 25 m with a JONSWAP spectrum defined by a significant wave height *H*_s_, peak period *T*_p_, and peak enhancement factor γ of 3.3. For all multihour model runs, the same 1-hour wave signal was used throughout. In all model runs, the reef platform was considered impermeable and immovable, and the island was permeable and moveable. The morphological response simulated by XBeach can be “sped up” through using the morphological acceleration factor (morfac) parameter. However, testing revealed that the island response strongly depended on the morfac value; therefore, morfac = 1 was used in all simulations. Default parameters were used in all simulations.

Seven different numerical modeling test series were conducted (Table 1), and each of these was a morphodynamic run (i.e., allowing for changing morphology). Test series XB1 consisted of a large number of numerical model runs with varying sediment size *D*_50_, hydraulic conductivity *K*, and sediment transport phase angle φ, representing the model validation for the physical experiment D3. Model performance was assessed through the BSS by comparing the modeled and measured morphological change

$$\text{BSS}=1-\frac{\frac{1}{n}\sum_{i=1}^{n}{\left(\Delta z_{\text{modeled},i}-\Delta z_{\text{measured},i}\right)}^{2}}{\frac{1}{n}\sum_{i=1}^{n}{\left(\Delta z_{\text{measured},i}\right)}^{2}}$$ (4)

where *n* is the number of observations (cross-shore positions) over which the morphological change occurred, and Δ*z*_modeled_ and Δ*z*_measured_ are the modeled and measured bed-level change, respectively. BBS ranges between 0 and 1, and the following qualifiers are generally used: <0.2 = poor, 0.2 to 0.5 = fair, 0.5 to 0.8 = good, and >0.8 = excellent. The modeled and measured elevation and position of the island crest were also compared. The performance of the numerical model is presented in Table 2, and the results of the “optimal” parameter settings are presented in Fig. 1.

**Table 1:** Hydrodynamic parameters used in numerical modeling. *z*_start_, profile at start of the simulation; *H*_s_, significant wave height; *T*_p_, wave period; *h*_reef_, water level on reef; SLR, sea level rise; *D*_50_, sediment size; *K*, hydraulic conductivity; φ, sediment transport phase angle; *T*_test_, test time.

**Test series**	***z*_start_**	***H*_s_ (m)**	***T*_p_ (s)**	***h*_reef_ (m)**	**SLR (m)**	***D*_50_ (mm)**	***K* (m s^−1^)**	**φ (°)**	***T*_test_ (hours)**
**XB1**	After D2	4	9.9	3	1	Variable	Variable	Variable	50
**XB2**	After D2	4	9.9	3	1	2–15	0.002–0.1	25–35	3
**XB3**	After D2	3	9.9	2–4	0–2	14	0.005	25	3
**XB4**	After D2	2–4	9.9	3	1	14	0.005	25	3
**XB5**	Actual (2013)	2.6/2.2	9.9	2→2.75	0→0.75	14	0.005	25	108
**XB6**	Actual (2013)	2.6/2.2 + >3 m	9.9	2→2.75	0→0.75	14	0.005	25	108
**XB7**	Actual (2013)	2.6/2.2	9.9	2→2.75	0→0.75	14	0.005	25	1620

**Table 2:** Results of the numerical model validation of the physical model run D3 (test series XB1). *D*_50_, sediment size; *K*, hydraulic conductivity; φ, sediment transport phase angle; BSS, Brier skill score (goodness of fit; cf. Eq. 4); Δ*z*_crest_, difference between modeled and measured island crest elevation; Δ*x*_crest_, difference between modeled and measured island crest position. For all model runs, *H*_s_ = 4 m, *T*_p_ = 9.9 s, *h*_reef_ = 3 m, and *T*_test_ = 50 hours. “X” denotes that the island was destroyed during the simulation. Bold values for BSS, Δ*z*_crest_, and Δ*x*_crest_ represent the best performance of the numerical model in each of the groups of model runs. Positive values for Δ*z*_crest_ and Δ*x*_crest_ mean that the modeled crest is higher and further landward, respectively.

**Test series**	***D*_50_ (mm)**	***K* (m s^−1^)**	**φ (°)**	**BSS (−)**	**Δ*z*_crest_ (m)**	**Δ*x*_crest_ (m)**
	**Sensitivity to sediment transport phase angle φ**					
**XB1_06_005_25**	6	0.005	25	X	X	X
**XB1_06_005_26**	6	0.005	27	X	X	X
**XB1_06_005_29**	6	0.005	29	0.79	0.06	14.0
**XB1_06_005_30**	6	0.005	30	**0.89**	**0.25**	8.2
**XB1_06_005_31**	6	0.005	31	0.86	0.40	4.9
**XB1_06_005_33**	6	0.005	33	0.74	0.60	**0.4**
**XB1_06_005_35**	6	0.005	35	0.63	0.74	−1.9
	**Sensitivity to hydraulic conductivity *K***					
**XB1_10_000_25**	10	0.000	25	0.53	−0.83	21.8
**XB1_10_005_25**	10	0.005	25	0.68	−0.56	17.0
**XB1_10_010_25**	10	0.010	25	**0.83**	**0.10**	6.2
**XB1_10_020_25**	10	0.020	25	0.66	0.42	**0.3**
**XB1_10_030_25**	10	0.030	25	0.50	0.54	−2.7
**XB1_10_040_25**	10	0.040	25	0.42	0.63	−4.3
**XB1_10_050_25**	10	0.050	25	0.36	0.71	−5.9
	**Sensitivity to sediment size *D***_**50**_					
**XB1_06_005_25**	6	0.005	25	X	X	X
**XB1_08_005_25**	8	0.005	25	X	X	X
**XB1_10_005_25**	10	0.005	25	0.68	−0.56	17.0
**XB1_12_005_25**	12	0.005	25	**0.88**	−0.20	9.7
**XB1_14_005_25**	14	0.005	25	0.77	**0.08**	**2.1**
	**Sensitivity to sediment size *D*_50_ and hydraulic conductivity *K***					
**XB1_06_010_25**	6	0.010	25	X	X	X
**XB1_07_020_25**	7	0.020	25	0.83	0.31	8.3
**XB1_08_030_25**	8	0.030	25	0.59	0.52	−**0.6**
**XB1_10_050_25**	10	0.050	25	0.36	0.71	−5.9
**XB1_12_070_25**	12	0.070	25	0.21	0.86	−7.3
**XB1_15_000_25**	15	0.000	25	**0.84**	**−0.17**	3.5
**XB1_15_050_25**	15	0.050	25	0.24	0.76	−7.4

Test series XB2, XB3, and XB4 were all 3-hour morphodynamic runs aimed at understanding the sensitivity of the reef island response to the crest overwash discharge and the balance between overtopping (crest accretion) and overwashing (crest lowering and island migration) under a range of forcing conditions. The results of these simulations are presented in Fig. 2. During test series XB2, model runs were carried out with constant extreme wave forcing (*H*_s_ = 3 m) and a 1-m SLR, and with each run consisting of a unique combination of sediment size *D*_50_ and hydraulic conductivity *K* as outlined in table S4. During test series XB3, sediment characteristics and wave forcing were kept constant (*D*_50_ = 14 mm, *K* = 0.005 m s^−1^, and *H*_s_ = 3 m) with a variable SLR (0 to 2 m); during test series XB4, sediment characteristics and sea level were kept constant (*D*_50_ = 14 mm, *K* = 0.005 m s^−1^, and SLR = 1 m) with a variable wave height (2 to 4 m). Overwash volumes at the crest were computed every 5 min, taking into account the moving crest location.

### SLR simulations

Three approaches were used to simulate how Fatato Island will adjust to a future SLR of 0.75 m, the global average sea level increase projected for 2100 under the IPCC (Intergovernmental Panel on Climate Change) scenario RCP8.5 (*23*): simulations XB5, XB6, and XB7 (Table 1). The same base wave condition was used for each simulation, informed by analysis of hindcast wave climate data and the island’s exposure to moderate-large waves at spring high tide conditions that promote maximum geomorphic change at the shoreline. The first approach was to increase sea level gradually from contemporary spring high tide (*h_reef_* = 2.0 m) by 0.75 m, with equivalent exposure to spring high tide and annual maximum wave heights that are predicted to occur between 2013 and 2100. Wave hindcast and tide gauge measurements show that wave heights exceeding 2.6 m [annual maximum wave height at Fatato; (*30*)] during spring tides [tides greater than 0.8 m above mean sea level (MSL)] occur for an average of 0.62 hour per year (over the past 34 years). Further analysis of this 30-year (1979–2013) wave hindcast using the generalized Pareto distribution reveals extreme wave heights of 2.51, 3.13, and 3.69 m for 1-, 10-, and 100-year return periods, respectively. The island’s exposure to annual maximum conditions at spring high tide (*H*_s_ = 2.6 m) can then be represented by exposing the island to a 54-hour spring high tide. To provide some relaxation from constant high energy, a 108-hour spectral JONSWAP boundary condition was developed, with *T*_s_ = 9.9 s and *H*_s_ alternating between 2.2 and 2.6 m each hour.

SLR simulation XB5 exposed the island to this 108-hour wave condition, with a linear increase in sea level of 0.75 m. SLR simulation XB6 used the exact same 108-hour wave time series as a base condition, but included “extreme” perturbations, where *H*_s_ increased for 3 hours, every 15 hours. The first extreme perturbation elevated wave height to *H*_s_ = 3.0 m, with each subsequent event associated with increasingly larger waves, until the final event of *H*_s_ = 3.8 m. These two 108-hour simulations represent an attempt to “geomorphically cheat time” and potentially do not provide sufficient exposure for the island to reach anything close to a morphodynamic equilibrium profile. Therefore, the third SLR simulation (XB7) exposed the island to the same 108-hour wave condition as in XB5 15 times, where sea level increased by 0.05 m every 108 hours (total simulation time of 1620 hours). This simulation does not represent a forecastable temporal duration but is aimed to provide insight into how the island morphology will adjust to a near equilibrium with small incremental increases in sea level, under moderate to high wave energy at spring high tide.

The same island profile was used at the start in all simulations. This initial profile was the output of a simulation where the 108-hour default boundary condition was used to “prime” the double-ridged island profile (surveyed in 2013) at contemporary spring high tide (*h_reef_* = 2.0 m). The primed island profile is characterized by a crest elevation 3.65 m above MSL or 4.65 m above the reef flat, with a spring high tide freeboard of 2.65 m. Calibrated input conditions of *D*_50_ = 14 mm, *K* = 0.005 m s^−1^, and φ = 25 were used in all SLR simulations.

## Supplementary Material

aay3656_SM.pdf

## SUPPLEMENTARY MATERIALS

Supplementary material for this article is available at http://advances.sciencemag.org/cgi/content/full/6/24/eaay3656/DC1
