# Supplementary material for: Coral reef islands can accrete vertically in response to sea level rise
Source: Sci Adv. 2020 Jun 10;6(24):eaay3656. doi: 10.1126/sciadv.aay3656 (PMC7286686; doi:10.1126/sciadv.aay3656)
Supplement: aay3656_SM.pdf [file aay3656_SM.pdf]

Science Advances
AAAS

advances.sciencemag.org/cgi/content/full/6/24/eaay3656/DC1

Supplementary Materials for

Coral reef islands can accrete vertically in response to sea level rise

Gerd Masselink*, Eddie Beetham, Paul Kench

*Corresponding author. Email: gerd.masselink@plymouth.ac.uk

Published 10 June 2020, *Sci. Adv.* **6**, eaay3656 (2020)
DOI: 10.1126/sciadv.aay3656

This PDF file includes:

Figs. S1 and S2
Tables S1 to S4

SUPPLEMENTARY MATERIALS

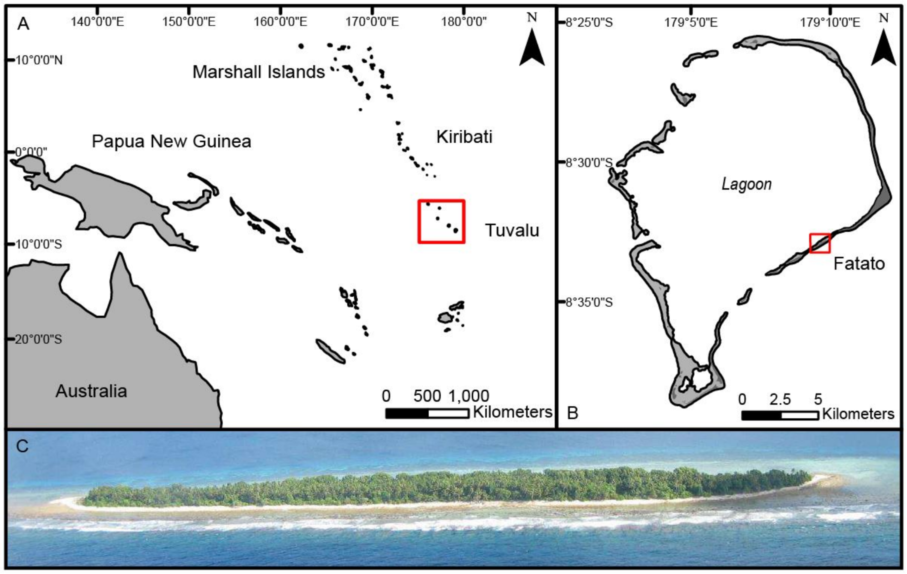

Figure S1. Environmental setting of Fatato Island. (a) Location of Tuvalu within the Pacific. (b) Location of Fatato Island, Funafuti atoll, Tuvalu. (c) Aerial photograph of Fatato Island taken by Paul Kench, February 2013.

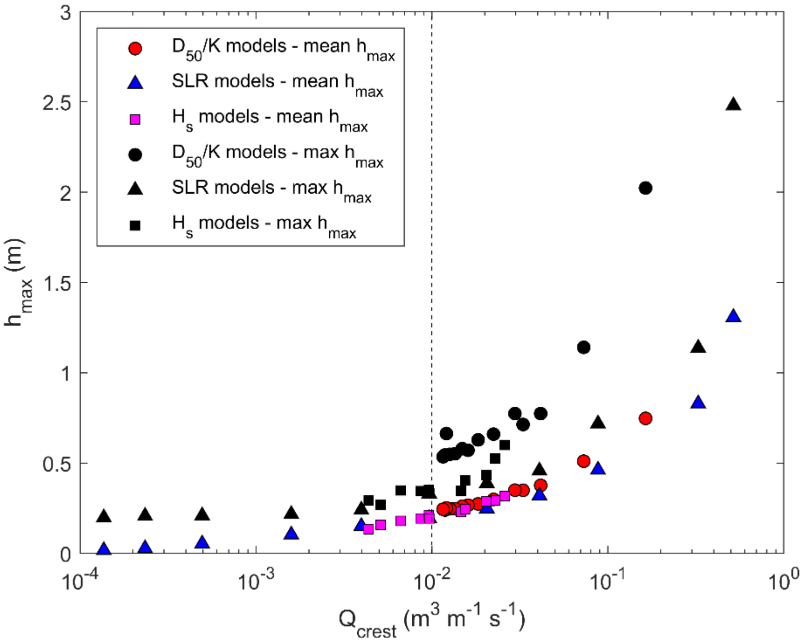

Figure S2. Relation between crest discharge and water depth during overwash. Comparison between mean overwash discharge across the island (Q_{crest}) and the maximum water depth during overwash at the island crest location (h_{max}) for the 3-hr numerical simulation shown in Figure 2. Two estimates for h_{max} are plotted: the maximum h_{max} attained over the 3-hr simulation period (usually attained at the end of the overwash simulations and at the start of the overtopping simulations) and the mean h_{max} computed as the average of 5-min h_{max} values.

Table S1. Summary of island morphology and overwash characteristics at the end of each SLR simulation. z_{crest} = crest elevation; Δz_{crest} = difference between initial and final island crest elevation; Δx_{crest} = difference between initial and final island crest position; $\Delta freeboard$ = reduction in freeboard (elevation difference between crest and sea level) relative to the start of the simulation; and Q_{crest} = overtopping discharge at the crest location. Hourly mean and maximum values for Q_{crest} are calculated for the final hour of each simulation at SLR = 0.75 m.

**Scenario**	z_{crest} **(m)**	Δz_{crest} **(m)**	Δx_{crest} **(m)**	$\Delta freeboard$ **(m)**	**Mean** Q_{crest} **(m^3 m^{-1} s^{-1})**	**Max** Q_{crest} **(m^3 m^{-1} s^{-1})**
**XB5**	4.929	0.284	9.0	-0.466	0.0025	0.4674
**XB6**	4.985	0.340	11.0	-0.410	0.0018	0.4185
**XB7**	5.321	0.676	16.4	-0.074	0.0003	0.2238

Table S2. Hydrodynamic parameters used within the Test Series A-C. *Test* = test series; H_s = significant wave height; T_p = peak wave period; and h_{reef} = water level on reef. The prototype values are given in brackets. The results shown in Fig. 1c, d, e relate to the mid-tide run with extreme wave conditions with a short wave period.

**Test Series**	H_s **(m)**			T_p **(s)**		h_{reef} **(m)**
	Energetic	*Storm*	*Extreme*	*Short*	*Long*	
**A (low tide)**	0.04 (2)	0.06 (3)	0.08 (4)	1.4 (9.9)	2.3 (16)	0(0)
**B (mid-tide)**	0.04 (2)	0.06 (3)	0.08 (4)	1.4 (9.9)	2.3 (16)	0.02(1)
**C (high tide)**	0.04 (2)	0.06 (3)	0.08 (4)	1.4 (9.9)	2.3 (16)	0.04(2)

Table S3. Hydrodynamic parameters used in physical flume experiments. z_{start} = profile at start of the simulation; H_s = significant wave height; T_p = wave period; h_{reef} = water level on reef; and T_{test} = test time. Prototype values are given in brackets.

**Test series**	Z_{start}	H_s **(m)**	T_p **(s)**	h_{reef} **(m)**	*SLR* **(m)**	D_{50} **(mm)**	T_{test} **(hrs)**
**D1**	Actual (2013)	0.08 (4)	1.4 (9.9)	0.04 (2)	0	0.3 (15)	1.5 (10.6)
**D2**	After D1	0.08 (4)	1.4 (9.9)	0.05 (2.5)	0.01 (0.5)	0.3 (15)	1.5 (10.6)
**D3**	After D2	0.08 (4)	1.4 (9.9)	0.06 (3)	0.02 (1)	0.3 (15)	7 (49.5)

Table S4. Relation between sediment size D_{50} **and hydraulic conductivity** K **used in numerical test series XB2.**

D_{50} **(mm)**	2	3	4	5	6	7	8	9	10	11	12	13	14	15
K **(mm s^{-1})**	2	4	6	8	10	20	30	40	50	60	70	80	90	100
